# Simultaneous inference for multiple marginal generalized estimating equation models

**DOI:** 10.1177/0962280219873005

**Published:** 2019-09-17

**Authors:** Robin Ristl, Ludwig Hothorn, Christian Ritz, Martin Posch

**Affiliations:** 1 Center for Medical Statistics, Informatics, and Intelligent Systems, Medical University of Vienna, Vienna, Austria; 2 Institute of Biostatistics, Leibniz University Hannover, Hannover, Germany; 3 Department of Nutrition, Exercise and Sports, Faculty of Science, University of Copenhagen, Copenhagen, Denmark

**Keywords:** Generalized estimating equations, multiple testing, multiple endpoints, dependent observations, small samples

## Abstract

Motivated by small-sample studies in ophthalmology and dermatology, we study the problem of simultaneous inference for multiple endpoints in the presence of repeated observations. We propose a framework in which a generalized estimating equation model is fit for each endpoint marginally, taking into account dependencies within the same subject. The asymptotic joint normality of the stacked vector of marginal estimating equations is used to derive Wald-type simultaneous confidence intervals and hypothesis tests for multiple linear contrasts of regression coefficients of the multiple marginal models. The small sample performance of this approach is improved by a bias adjustment to the estimate of the joint covariance matrix of the regression coefficients from multiple models. As a further small sample improvement a multivariate *t*-distribution with appropriate degrees of freedom is specified as reference distribution. In addition, a generalized score test based on the stacked estimating equations is derived. Simulation results show strong control of the family-wise type I error rate for these methods even with small sample sizes and increased power compared to a Bonferroni-Holm multiplicity adjustment. Thus, the proposed methods are suitable to efficiently use the information from repeated observations of multiple endpoints in small-sample studies.

## 1 Introduction

In empirical studies where for each subject multiple endpoints are observed, it is often of interest to identify predictive factors for several of these endpoints. To this end, regression models for the different endpoints can be defined to test respective null hypotheses on the model parameters. However, if for each endpoint, one (or more) hypotheses are tested, a multiple testing problem arises and adjustments for multiplicity are required to control, for example, the family wise type I error rate (FWER).

In this manuscript, we focus on settings where all or some of the multiple endpoints are measured repeatedly and derive multiple testing procedures and simultaneous confidence intervals that account for the correlation between the endpoints as well as the correlation between the repeated measurements of each endpoint. The endpoints may be on different scales (we particularly consider continuous, binary and count data), and the regression models may differ across endpoints. The proposed tests improve the Bonferroni test which is typically strictly conservative.

The testing procedures are based on generalized estimating equation (GEE) models^1^ that are separately fitted for each endpoint. Thereby each model accounts for the dependencies between repeated observations of the according endpoint.

We use a representation of stacked estimating equations to show joint multivariate asymptotic normality of the regression coefficient estimators from different models and to estimate their covariance matrix, such that parametric inference methods based on a multivariate normal approximation can be applied. The method generalizes the approach by Pipper et al.^2^ who used stacked estimating equations for multiple generalized linear models and Rochon^3^ who studied the case of repeated bivariate measurements comprising a continuous and a binary endpoint. Jensen et al.^4^ applied a similar representation to multiple linear mixed models for repeatedly observed continuous endpoints. Also see Verbeke et al.^5^ for a recent review on the analysis of multivariate longitudinal data.

While inference based on the multivariate normal approximation can be justified for large sample sizes by asymptotic arguments,^6^ it may be inaccurate for smaller samples. In particular, the bias and variability of nuisance parameter estimates are neglected in purely asymptotic methods, resulting in too liberal inference procedures. To improve the small sample properties of Wald tests, we generalize bias-adjustment procedures proposed for covariance matrix estimators in single GEE models,^7^ to the case of multiple marginal GEE models. Furthermore, similar to the studies by Hasler and Hothorn^8^ and Pan and Wall,^9^ we use multivariate *t*- and *F*-distributions to better control the type I error rate. We further propose a maximum-type generalized score test and show via simulation that it is a viable small sample alternative to the Wald test.

The paper is structured as follows: In Section 2, multiple marginal GEE models and a bias-adjusted covariance matrix estimator are introduced. In Section 3, we define Wald and score test statistics to test multiple linear contrasts and derive corresponding simultaneous confidence intervals. In Section 4, the proposed methods are applied to a retina disease study. Furthermore, in Section 5, we investigate the small sample properties of the proposed methods in a simulation study. Finally, in Section 6, we conclude with a discussion.

## 2 Multiple marginal GEE models

### 2.1 The statistical model

Assume that multiple endpoints (outcome variables) indexed by *m* = 1,...,*M* are observed in subjects with index *i* = 1,...,*K*. Observations between different subjects are assumed to be independent. However, we allow for repeated observations, indexed by $j=1,\ldots ,n_{i^{\left(m\right)}}$, of the *m*-th endpoint within subjects such that $Y_{\mathrm{ij}}^{\left(m\right)}$ denotes the *j*-th observation of endpoint *m* in subject *i*. Let $x_{\mathrm{ij}}^{\left(m\right)}$ be a row-vector of covariates that is of length $p^{\left(m\right)}$ and $\beta^{\left(m\right)}$ a vector of regression coefficients.

Each endpoint *m* = 1 ,…, *M* is modeled with a separate generalized linear regression model for the mean $\mu_{\mathrm{ij}}^{\left(m\right)}=E(Y_{\mathrm{ij}}^{\left(m\right)})={g^{\left(m\right)}}^{-1}(x_{\mathrm{ij}}^{\left(m\right)}\beta^{\left(m\right)})$ with link function $g^{\left(m\right)}$, where the variance of $Y_{\mathrm{ij}}^{\left(m\right)}$ is modeled as $\mathrm{var}(Y_{\mathrm{ij}}^{\left(m\right)})=\nu (\mu_{\mathrm{ij}}^{\left(m\right)})\varphi^{\left(m\right)}$, where *ν* is a variance function depending only on $\mu_{\mathrm{ij}}^{\left(m\right)}$ and $\varphi^{\left(m\right)}$ is a scale parameter. In typical applications, the link function and the variance function are derived from the canonical representation of an exponential family model.^10^ Throughout the manuscript, we assume that regression coefficients and nuisance parameters are unique to one model and not shared between any two models. The models for different endpoints are estimated independently, see Section 7 for a discussion on alternative approaches of joint estimation.

### 2.2 Generalized estimating equations

To account for dependencies between repeated observations within the same subject, the regression coefficients $\beta^{\left(m\right)}$ and their covariance matrix are estimated based on the generalized estimating equation approach.^1^ Thus, the estimate ${\overset{\wedge }{\beta }}^{\left(m\right)}$ is given by the solution of the generalized estimating equation

(1) $$U^{\left(m\right)}(\beta^{\left(m\right)})=\sum_{i=1}^{K}U_{i}^{\left(m\right)}(\beta^{\left(m\right)})=0$$

with subject-wise contributions ${\text{U}}_{i}^{\left(m\right)}={\text{D}}_{i}^{\left(m\right)}{\;}^{T}{\text{V}}_{i}^{\left(m\right)}{\;}^{-1}{\text{S}}_{i}^{\left(m\right)}$. Here, ${\text{S}}_{i}^{\left(m\right)}={\text{Y}}_{i}^{\left(m\right)}-{\text{μ}}_{i}^{\left(m\right)}$ is a vector of residuals. ${\text{V}}_{i}^{\left(m\right)}={\text{A}}_{i}^{\left(m\right)}{\;}^{1/2}{\text{R}}_{i}^{\left(m\right)}(\text{α}){\text{A}}_{i}^{\left(m\right)}{\;}^{1/2}\phi^{\left(m\right)}$ is a working covariance matrix with ${\text{A}}_{i}^{\left(m\right)}=diag(\nu (\mu_{ij}^{\left(m\right)}))$ the diagonal matrix of variance functions and $R_{i}^{\left(m\right)}$ the working correlation of $Y_{i}^{\left(m\right)}$. $R_{i}^{\left(m\right)}$ is parametrized via a parameter vector $\alpha^{\left(m\right)}$ (which typically is of small dimension compared to the number of entries in $R^{\left(m\right)}$). ${\text{D}}_{i}^{\left(m\right)}={\left(\frac{\partial {\text{μ}}_{i}^{\left(m\right)}}{\partial {\text{β}}^{\left(m\right)}}\right)}^{T}$, and in an exponential family model with canonical link ${\text{D}}_{i}^{\left(m\right)}{\;}^{T}=\frac{\partial {\text{μ}}_{i}^{\left(m\right)}}{\partial {\text{β}}^{\left(m\right)}}={\text{X}}_{i}^{\left(m\right)}{\;}^{T}{\text{A}}_{i}^{\left(m\right)}$, with ${\text{X}}_{i}^{\left(m\right)}{\;}^{T}=\left({\text{x}}_{i1}^{\left(m\right)}{\;}^{T},\ldots ,{\text{x}}_{in_{i}}^{\left(m\right)}{\;}^{T}\right)$.

Given $\beta^{\left(m\right)}$, the parameters $\alpha^{\left(m\right)}$ and $\varphi^{\left(m\right)}$ may be consistently estimated from the residuals $S_{i}^{\left(m\right)},i=1,\ldots ,K$ by moment estimators.^1^ Given $\alpha^{\left(m\right)}$ and $\varphi^{\left(m\right)}$, an estimate for $\beta^{\left(m\right)}$ is found as solution to equation (1). Iteration of these two estimation steps results in a consistent estimate ${\overset{\wedge }{\beta }}^{\left(m\right)}$ such that asymptotically $K^{1/2}({\overset{\wedge }{\beta }}^{\left(m\right)}-\beta^{\left(m\right)})$ is multivariate normal (Theorem 2 in Liang and Zeger^1^). A consistent estimator for the covariance matrix of the limiting normal distribution as proposed in Liang and Zeger^1^ is $(\frac{1}{K}{\overset{\wedge }{H}}^{\left(m\right)}{)}^{-1}\frac{1}{K}{\overset{\wedge }{B}}^{\left(m\right)}(\frac{1}{K}{\overset{\wedge }{H}}^{\left(m\right)}{)}^{-1}$. Here, ${\hat{\text{B}}}^{\left(m\right)}=\sum_{i=1}^{K}{\text{U}}_{i}^{\left(m\right)}{\text{U}}_{i}^{\left(m\right)}{\;}^{T}$ and ${\hat{\text{H}}}^{\left(m\right)}=-\sum_{i=1}^{K}{\text{D}}_{i}^{\left(m\right)}{\;}^{T}{\text{V}}_{i}^{\left(m\right)}{\;}^{-1}{\text{D}}_{i}^{\left(m\right)}$ both evaluated at ${\overset{\wedge }{\beta }}^{\left(m\right)}$, with $\frac{1}{K}{\hat{\text{H}}}^{\left(m\right)}$ converging to $\frac{1}{K}{\text{H}}^{\left(m\right)}=\frac{1}{K}\frac{\partial {\text{U}}^{\left(m\right)}({\text{β}}^{\left(m\right)})}{\partial {\text{β}}^{\left(m\right)}}$. The asymptotic results for ${\overset{\wedge }{\beta }}^{\left(m\right)}$ do not require that the working correlation $R_{i}$ matches the true correlation of $Y_{i}$; however, the efficiency of $\overset{\wedge }{\beta }$ increases if $R_{i}$ is close to the true correlation.

Note that in case the mean model is misspecified, $\overset{\wedge }{\&}\beta^{\left(m\right)}$ will typically converge to a vector ***β***^(*m*)^ that defines the model within the chosen mean structure that best approximates the true model in the sense of minimized Kullback-Leibler distance.^11,12^ In that case, the proposed methods provide inference on the parameters of the approximating model.

### 2.3 Multiple marginal models

We are interested in simultaneous inference on the regression coefficient vectors $\beta^{\left(1\right)},\ldots ,\beta^{\left(M\right)}$ and approximate the joint distribution of the stacked vector $\overset{\wedge }{\beta }=({\overset{\wedge }{\beta }}^{(1)T},\ldots ,{\overset{\wedge }{\beta }}^{(M)T}){\&}^{T}$ by a multivariate normal distribution based on the framework of Pipper et al.^2^

By equation (1), $\overset{\wedge }{\beta }$ (together with the marginal model estimates for the nuisance parameters $\alpha^{\left(m\right)}$ and $\varphi^{\left(m\right)},m=1,\ldots ,M$) is the solution to the stacked estimating equation

(2) $$\begin{array}{c} U=(\begin{array}{c} U^{\left(1\right)}\\ :\\ U^{\left(M\right)} \end{array})=\sum_{i=1}^{K}(\begin{array}{c} U_{i}^{\left(1\right)}\\ :\\ U_{i}^{\left(M\right)} \end{array})=\sum_{i=1}^{K}U_{i}=0 \end{array}$$

Similar to the case of a single GEE model, for increasing number of subjects *K*, $\sqrt{K}(\overset{\wedge }{\beta }-\beta )$ converges to a multivariate normal distribution with mean zero and covariance matrix $\lim_{p}(\frac{1}{K}\frac{\partial U(\beta )}{\partial \beta }{)}^{-1}(\frac{1}{K}\sum_{i=1}^{K}U_{i}(\beta )U_{i}{\left(\beta \right)}^{T})(\frac{1}{K}\frac{\partial U(\beta )}{\partial \beta }{)}^{-1}$ provided $\overset{\wedge }{\beta }$ is consistent for β and certain regularity conditions are met. Here *lim*_*p*_ denotes the limit in probability when *K* goes to infinity. Consistency of $\overset{\wedge }{\beta }$ follows if ${\overset{\wedge }{\beta }}^{\left(m\right)}$ is consistent for $\beta^{\left(m\right)}$ for all *m* = 1,…,*M*. The essential regularity conditions concern the derivatives of U with respect to β (see Chapter 5.3 in the book by Van der Vaart^6^ and Chapter 9.1 in the book by Cox and Hinkley^13^). However, the matrix of first derivatives $H=\frac{\partial U(\beta )}{\partial \beta }$ is a block diagonal matrix of the matrices $H^{\left(m\right)}=\frac{\partial U^{\left(m\right)}(\beta^{\left(m\right)})}{\partial \beta^{\left(m\right)}}$. Hence, conditions such as existence of derivatives, a dominating function, expectation and a matrix-inverse are inherited if they are met by all marginal models.

An estimate of the covariance matrix of $\overset{\wedge }{\beta }$ is given by

(3) $$\begin{array}{c} \overset{\wedge }{\Sigma }={\overset{\wedge }{H}}^{-1}\overset{\wedge }{B}{\overset{\wedge }{H}}^{-1}=(\begin{array}{ccc} {\overset{\wedge }{H}}^{(1)-1}{\overset{\wedge }{B}}^{\left(1,1\right)}{\overset{\wedge }{H}}^{(1)-1} & \ldots & {\overset{\wedge }{H}}^{(1)-1}{\overset{\wedge }{B}}^{\left(1,M\right)}{\overset{\wedge }{H}}^{(M)-1}\\ : & :\\ {\overset{\wedge }{H}}^{(1)-1}{\overset{\wedge }{B}}^{\left(M,1\right)}{\overset{\wedge }{H}}^{(M)-1} & \ldots & {\overset{\wedge }{H}}^{(M)-1}{\overset{\wedge }{B}}^{\left(M,M\right)}{\overset{\wedge }{H}}^{(M)-1} \end{array}) \end{array}$$

where $\overset{\wedge }{B}=\sum_{i=1}^{K}U_{i}(\overset{\wedge }{\beta })U_{i}(\overset{\wedge }{\beta }){\&}^{T}$ is calculated from the stacked vectors $U_{i}$. The components ${\overset{\wedge }{B}}^{\left(m,m'\right)}=\sum_{i=1}^{K}U_{i}^{\left(m\right)}({\overset{\wedge }{\beta }}^{\left(m\right)})U_{i}^{m'}({\overset{\wedge }{\beta }}^{\left(m'\right)}){\&}^{T}$ correspond to the empirical correlation between the contributions of a subject to the estimating equations of models m,m'. $\overset{\wedge }{H}$ is a block diagonal matrix with block elements ${\overset{\wedge }{H}}^{\left(m\right)}=\frac{\partial U^{\left(m\right)}({\overset{\wedge }{\beta }}^{\left(m\right)})}{\partial \beta^{\left(m\right)}}$ that are the corresponding estimates from the marginal models. The resulting multiple model sandwich variance estimator maintains the marginal GEE sandwich variance estimators in the diagonal blocks, while the off diagonal blocks contain estimates for the covariances between estimated regression coefficients from different models.

#### 2.3.1 Bias-adjusted covariance estimator

The covariance matrix estimator in GEE models is consistent but it is in general not unbiased. With small sample sizes, the variances may be underestimated which leads to an inflation of the type I error rate of hypothesis tests and to confidence intervals with coverage less than the nominal 1 – *α* level. Based on the bias-adjusted estimator proposed by Mancl and DeRouen^7^ (see also Wang et al.^14^) for a single GEE model, we derive a bias-adjusted covariance estimator for multiple models, given by

(4) $${\overset{\wedge }{\Sigma }}_{\mathrm{adj}}={\overset{\wedge }{H}}^{-1}{\overset{\wedge }{B}}_{\mathrm{adj}}{\overset{\wedge }{H}}^{-1}$$

where ${\overset{\wedge }{B}}_{\mathrm{adj}}=\sum_{i=1}^{K}D_{i}^{T}V_{i}^{-1}(I_{i}-{\overset{\wedge }{P}}_{\mathrm{ii}}){\&}^{-1}S_{i}S_{i}^{T}(I_{i}-{\overset{\wedge }{P}}_{\mathrm{ii}}){\&}^{-1}V_{i}^{-1}D_{i}$ and ${\overset{\wedge }{P}}_{\mathrm{ii}}=D_{i}{\overset{\wedge }{H}}^{-1}D_{i}^{T}V_{i}^{-1}$ and $I_{i}$ is the identity matrix with matching dimension. See the supplemental material Section S.1 for details. The matrices $D_{i}$, $V_{i}$ and, consequently, ${\overset{\wedge }{P}}_{\mathrm{ii}}$ are block diagonal with block elements $D_{i}^{\left(m\right)},V_{i}^{\left(m\right)}$, ${\overset{\wedge }{P}}_{\mathrm{ii}}^{\left(m\right)},m=1,\ldots ,M$, respectively. ${\overset{\wedge }{\Sigma }}_{\mathrm{adj}}$ contains in the diagonal blocks the bias-adjusted variance matrix estimators that would result from separate marginal models and the off-diagonal blocks contain bias-adjusted estimates of the covariances between regression coefficient estimates from different models.

### 2.4 Missing data

Values of some $Y_{\mathrm{ij}}^{\left(m\right)}$ or $x_{\mathrm{ij}}^{\left(m\right)}$ may be missing in an actual data set. As for single GEE models,^1^ $\overset{\wedge }{\beta }$ is consistent for β if some observations are missing completely at random (MCAR), i.e. missingness is completely independent from any missing or non-missing values of the included variables, and each model is fit with the respective available data. Also, inference based on the asymptotic normality of the stacked score vector U stays unaffected under MCAR. If a subject *i* has to be excluded entirely from the model for the *m*-th endpoint due to missing data, the realization of the respective contribution to the estimating equation is treated as $U_{i}^{\left(m\right)}=0$ in equations (1) and (2) and subsequent calculations. Note that this does not bias the estimated covariance matrix (3) or (4) as the effective sample size enters these equations in terms of the observed information ${\overset{\wedge }{H}}^{-1}$ and not the number of clusters.

If data are missing at random (MAR), i.e. the missingness may depend on non-missing values of observed variables, residuals may be biased. Consequently, a model fit with available data may result in biased and inconsistent estimates. This bias may be counteracted by weighing observed residuals with the inverse probability of non-missingness of the given data point. Under MAR, these probabilities may in principle be estimated from the observed data. There are different ways to introduce weights in the generalized estimating equation.^15–18^ Our software implementation, discussed in Section 6, allows for weights that resemble a scale factor for each observation, similar to the GENMOD procedure in SAS.^19^ Here, subject-wise contributions to a correspondingly weighted generalized estimating equation are of the form ${\text{D}}_{i}^{\left(m\right)}{\;}^{T}{\text{W}}_{i}^{1/2}{\text{V}}_{i}^{\left(m\right)}{\;}^{-1}{\text{W}}_{i}^{1/2}{\text{S}}_{i}^{\left(m\right)}$, where $W_{i}$ is a diagonal matrix of weights for the observations of subject *i*. When all observations of a subject receive the same weight, this formulation is equivalent to the cluster-weighted GEE proposed by Fitzmaurice and Laird.^15^ With an identity working correlation, it is equivalent to the weighted GEE proposed by Robins et al.^16^

## 3 Maximum-type tests and quadratic form tests for linear hypotheses

Consider a null hypothesis of the form $H_{0}:L\beta -r=0$, where L is a matrix of linear constraints with *c* rows and number of columns equal to the length of β and r is a vector of matching dimension. Each row of this equation corresponds to an elementary null hypothesis $H_{i}:(L\beta ){\&}_{i}=r_{i},i=1,\ldots ,c$. Furthermore, assume that Lβ=r has at least one solution in β. In Section 3.1, we construct asymptotic hypothesis tests for the global null hypothesis *H*_0_ that are based on the maximum of multivariate Wald statistics, and we propose adjustments of the tests for small samples. In Section 3.2, a maximum test based on score statistics is proposed. In Section 3.3, we discuss Wald and score tests for *H*_0_ that are based on quadratic forms. In Section 3.4, the closed testing principle is applied to construct multiple testing procedures, allowing for decisions on intersection and elementary hypotheses with type I error rate control. Furthermore, simultaneous confidence intervals for $(L\beta ){\&}_{i},i=1,\ldots ,c$ corresponding to a single step multiple testing procedure are derived.

### 3.1 Maximum-type Wald test

The maximum test rejects *H*_0_ if

$$\max_{i=1,\ldots ,c}|((L\overset{\wedge }{\beta }{)}_{i}-r_{i})/{\overset{\wedge }{\mathrm{SE}}}_{i}|>q_{1-\alpha }$$

where we use the normal approximation $\sqrt{K}(L\overset{\wedge }{\beta }-L\beta )\approx N(0,KL\overset{\wedge }{\Sigma }L^{T})$ to define the critical value $q_{1-\alpha }$ as the solution of $P(\max_{i=1,\ldots ,c}|Z_{i}|\leq q_{1-\alpha })=1-\alpha$.^20^ Here, Z denotes a *c*-dimensional multivariate normal variable with mean 0, unit variances and correlation structure given by $L\overset{\wedge }{\Sigma }L^{T}$, such that $\mathrm{cov}(Z)=\mathrm{diag}(\overset{\wedge }{\mathrm{SE}}){\&}^{-1}L\overset{\wedge }{\Sigma }L^{T}\mathrm{diag}(\overset{\wedge }{\mathrm{SE}}){\&}^{-1}$, where the vector of standard errors $\overset{\wedge }{\mathrm{SE}}$ is given by the square roots of the diagonal entries of $L\overset{\wedge }{\Sigma }L^{T}$. Similarly, the p-value for the maximum test is defined as $P(\max_{i=1,\ldots ,c}|Z_{i}|\geq \max_{i=1,\ldots ,c}|((L\overset{\wedge }{\beta }){\&}_{i}-r_{i})/{\overset{\wedge }{\mathrm{SE}}}_{i}|)$.

L may be below full rank since the quantile $q_{1-\alpha }$ is also defined for a degenerate multivariate normal distribution with singular covariance matrix.

#### 3.1.1 Small sample improvements

For small samples, the type I error rate of the above test can be considerably greater than the nominal level. As a first small sample improvement, the bias-adjusted covariance estimate ${\overset{\wedge }{\Sigma }}_{\mathrm{adj}}$ (equation (4)) may be used instead of $\overset{\wedge }{\Sigma }$ (equation (3)). Furthermore, to also account for the variability of the covariance estimators, the critical value $q_{1-\alpha }$ of the multivariate normal distribution can be replaced by the critical value $t_{1-\alpha }$ of a multivariate *t*-distribution,^21^ such that $P(\max_{i=1,\ldots ,c}|T_{i}|\leq t_{1-\alpha })=1-\alpha$, where T is distributed according to a *c*-dimensional multivariate *t*-distribution with correlation matrix $\mathrm{diag}(\overset{\wedge }{\mathrm{SE}}){\&}^{-1}L\overset{\wedge }{\Sigma }L^{T}\mathrm{diag}(\overset{\wedge }{\mathrm{SE}}){\&}^{-1}$ and an appropriate number of degrees of freedom (*df*). See the earlier studies^7,8,22^ for related approaches in the context of multiple contrast tests. As a simple method to choose the error degrees of freedom, we propose $\mathrm{df}=\min_{m=1,\ldots ,M}(K-p^{\left(m\right)})$ where $p^{\left(m\right)}$ is the number of regression coefficients in model *m* (compare with Munzel and Hothorn^23^). Alternative methods to choose degrees of freedom for multivariate comparisons are discussed in Section 7.

### 3.2 Maximum-type score test

We derive the maximum-type generalized score test as an approximation to the Wald test. By first order approximation, $\text{L}\hat{\text{β}}-\text{r}=\text{L}\hat{\text{β}}-\text{Lβ}\approx -\text{L}{\text{H}}^{-1}\text{U}(\text{β})$. Hence, tests for *H*_0_ can be constructed based on the right hand side $-LH^{-1}U(\beta )$ and its normal approximation under the null hypothesis, $N(0,LH^{-1}BH^{-1}L^{T})$. Under a simple null hypothesis, the true β is known, under a composite null hypothesis, a restricted estimate $\overset{∼}{\beta }$, which satisfies $L\overset{∼}{\beta }-r=0$, is plugged in. To estimate the limiting distribution covariance matrix, H and B are replaced by estimates $\tilde{\text{H}}=-{\text{D}}_{i}^{\left(m\right)}{\;}^{T}{\text{V}}_{i}^{\left(m\right)}{\;}^{-1}{\text{D}}_{i}^{\left(m\right)}$ evaluated at ${\overset{∼}{\beta }}^{\left(m\right)}$ and $\tilde{\text{B}}=\sum_{i=1}^{K}{\text{U}}_{i}(\tilde{\text{β}}){\text{U}}_{i}{\left(\tilde{\text{β}}\right)}^{T}$.

The maximum-type score test rejects *H*_0_ if

$$\max_{i=1,\ldots ,c}|(L{\overset{∼}{H}}^{-1}U(\overset{∼}{\beta })){\&}_{i}/{\overset{∼}{\mathrm{SE}}}_{i}|>{\overset{∼}{q}}_{1-\alpha }$$

Here ${\overset{∼}{\mathrm{SE}}}_{i}$ is the square root of the *i*-th diagonal element of $L{\overset{∼}{H}}^{-1}\overset{∼}{B}{\overset{∼}{H}}^{-1}L^{T}$. ${\overset{∼}{q}}_{1-\alpha }$ satisfies $P(\max_{i=1,\ldots ,c}|{\overset{∼}{Z}}_{i}|\leq {\overset{∼}{q}}_{1-\alpha })=1-\alpha$, where $\overset{∼}{Z}$ denotes a *c*-dimensional multivariate normal variable with mean 0, and covariance matrix given by $\mathrm{diag}(\overset{∼}{\mathrm{SE}}){\&}^{-1}L{\overset{∼}{H}}^{-1}\overset{∼}{B}{\overset{∼}{H}}^{-1}L^{T}\mathrm{diag}(\overset{∼}{\mathrm{SE}}){\&}^{-1}$.

For a single marginal GEE model, the restricted estimate ${\overset{∼}{\beta }}^{\left(m\right)}$ can be computed by the iterative restricted weighted least squares algorithm ${\overset{∼}{\beta }}^{\left(m,j+1\right)}={\overset{∼}{\beta }}^{\left(m,j\right)}-(\frac{\partial U}{\partial \beta }({\overset{∼}{\beta }}^{\left(m,j\right)}){)}^{-1}(U({\overset{∼}{\beta }}^{\left(m,j\right)})-L^{T}\lambda^{\left(j\right)})$, with the vector of Lagrange multipliers $\lambda^{\left(j\right)}=-(L(\frac{\partial U}{\partial \beta }({\overset{∼}{\beta }}^{\left(m,j\right)}))-1L^{T}){\&}^{-1}(L{\overset{∼}{\beta }}^{\left(m,j\right)}-r-L(\frac{\partial U}{\partial \beta }({\overset{∼}{\beta }}^{\left(m,j\right)}))-1U({\overset{∼}{\beta }}^{\left(m,j\right)}))$, compare with Rao and Toutenburg.^24^ Here, the second superscript indicates the iteration number. Where $\overset{\wedge }{\beta }$ can be understood to maximize a quasi-likelihood with first derivative $U,\overset{∼}{\beta }$ maximizes the quasi-likelihood subject to the restriction of *H*_0_.

If the null hypothesis Lβ-r=0 has a block diagonal structure such that each constraint involves only parameters of one marginal model, we have

(5) $$\begin{array}{c} L\beta =(\begin{array}{c} L^{\left(1\right)}\\ L^{\left(M\right)} \end{array})(\begin{array}{c} \beta^{\left(1\right)}\\ :\\ \beta^{\left(M\right)} \end{array})=(\begin{array}{c} L^{\left(1\right)}\beta^{\left(1\right)}\\ L^{\left(M\right)}\beta^{\left(M\right)} \end{array}) \end{array}$$

Then, the restricted estimate $\overset{∼}{\beta }$ is a stacked vector of restricted estimates from the marginal models. We consider only null hypotheses covered by equation (5). Otherwise, the elements of $\overset{∼}{\beta }$ needed to be estimated jointly for all models. However, contrasts between coefficients from different marginal models are rarely of interest, if they correspond to different units or scales of measurement. If outcomes are indeed measured at the same scale and units, they can be modelled together in one GEE model.

#### 3.2.1 Small sample considerations

With the score test, nuisance parameters are estimated under the null hypothesis based on the restricted estimate $\overset{∼}{\beta }$, which is less variable than $\overset{\wedge }{\beta }$. (In the limit $\mathrm{cov}(\sqrt{K}(\overset{∼}{\beta }-\beta )=\mathrm{cov}(\sqrt{K}(\overset{\wedge }{\beta }-\beta )-H_{p}^{-1}L^{T}({\mathrm{LH}}_{p}^{-1}L^{T}){\&}^{-1}LH_{p}$, where $H_{p}=\mathrm{li}m_{p}\frac{1}{K}H$.) Consequently, we may expect that the nuisance parameters are estimated with less variability, too, and the type I error rate control with the score test is improved compared to the unadjusted Wald test. In principle, though, the Mancl and DeRouen bias adjustment can be extended to the estimate $\overset{∼}{B}$. Instead of ${\overset{\wedge }{P}}_{\mathrm{ii}}=D_{i}{\overset{\wedge }{H}}^{-1}D_{i}^{T}V_{i}^{-1}$, the adjustment utilizes ${\overset{∼}{P}}_{\mathrm{ii}}=D_{i}(I-{\overset{∼}{H}}^{-1}L^{T}(L{\overset{∼}{H}}^{-1}L^{T}){\&}^{-1}L){\overset{∼}{H}}^{-1}D_{i}^{T}V_{i}^{-1}$. When calculating the score test, $\overset{∼}{B}$ is replaced by ${\overset{∼}{B}}_{\mathrm{adj}}=\sum_{i=1}^{K}D_{i}^{T}V_{i}^{-1}(I_{i}-{\overset{∼}{P}}_{\mathrm{ii}}){\&}^{-1}S_{i}S_{i}^{T}(I_{i}-{\overset{∼}{P}}_{\mathrm{ii}}){\&}^{-1}V_{i}^{-1}D_{i}$. See supplemental material Section S.1 for the derivation. Also, a multivariate *t* reference distribution could be used. We will, however, focus on the unadjusted score test in the numeric simulations.

### 3.3 Quadratic form tests

Alternatives to the maximum-type tests may be derived based on the normal approximation of the multivariate Wald and score statistics. An example are quadratic form tests. The quadratic form Wald test rejects *H*_0_ if

$$(L\overset{\wedge }{\beta }-r{)}^{T}(L\overset{\wedge }{\Sigma }L^{T}{)}^{-1}(L\overset{\wedge }{\beta }-r)>Q_{c}^{\left(\chi^{2}\right)}(1-\alpha )$$

where $Q_{c}^{\left(\chi^{2}\right)}(1-\alpha )$ denotes the 1 – *α* quantile of the chi-squared distribution with *c* degrees of freedom.

As small sample improvement, $\overset{\wedge }{\Sigma }$ may be replaced by ${\overset{\wedge }{\Sigma }}_{\mathrm{adj}}$. Further, $Q_{c}^{\left(\chi^{2}\right)}(1-\alpha )$ may be replaced by ${\mathrm{cQ}}_{c,\mathrm{df}}^{\left(F\right)}(1-\alpha )$ or $\frac{\mathrm{df}}{\mathrm{df}-c+1}{\mathrm{cQ}}_{c,\mathrm{df}-c+1}^{\left(F\right)}(1-\alpha )$, where $Q_{c,\mathrm{df}}^{\left(F\right)}(1-\alpha )$ denotes the 1 – *α* quantile of an *F*-distribution with *c* numerator degrees of freedom and *df* denominator degrees of freedom, chosen in the same way as for the maximum test. The former option is analogous to an *F*-test, where the variability of an assumedly independent and chi-squared distributed single nuisance parameter is taken into account. The latter option is analogous to Hotelling's test which adjusts for the variability of an assumedly independent and Wishart distributed covariance matrix estimate. This approach was described by Kenward and Roger^25^ for random effects models and by Pan and Wall^9^ in the context of single GEE models.

The quadratic form generalized score test rejects *H*_0_ if

$$U(\overset{∼}{\beta }){\&}^{T}{\overset{∼}{H}}^{-1}L^{T}(L{\overset{∼}{H}}^{-1}\overset{∼}{B}{\overset{∼}{H}}^{-1}L^{T}{)}^{-1}L{\overset{∼}{H}}^{-1}U(\overset{∼}{\beta })>Q_{c}^{\left(\chi^{2}\right)}(1-\alpha )$$

Similar to the maximum score test, this test is based on the normal approximation for the statistic $-L{\overset{∼}{H}}^{\left(-1\right)}U(\overset{∼}{\beta })$. For an alternative derivation see the study by Boos.^26^

If *L* is below full rank, a generalized matrix inverse may be applied in the calculation of the quadratic form statistics and *c* is replaced by the rank of *L*.

In terms of the multivariate space of $L\overset{\wedge }{\beta }-r$, the quadratic form test statistic and the maximum test statistic apply different metrics to measure deviations from the null vector. The quadratic form test is monotone in the non-centrality parameter $\left(L\beta -r){\&}^{T}(L\Sigma L^{T}){\&}^{-1}(L\beta -r\right)$; however, it is in general not monotone in the observed effects $\left|(L\overset{\wedge }{\beta }){\&}_{i}\right|$. In contrast, the maximum test is monotone in $\left|(L\overset{\wedge }{\beta }){\&}_{i}\right|$. In the setting of single regression models, a familiar application of quadratic form tests is testing a null hypothesis of equal effects between all, *c* + 1 say, stages of some grouping variable. There, the elements of $L\overset{\wedge }{\beta }$ constitute a set of *c* arbitrarily selected between-group differences and a quadratic metric is appropriate to assess the overall deviation from the null hypothesis. If, however, the null hypothesis refers to a set of effects in multiple models, each elements of $L\overset{\wedge }{\beta }$ has an individual interpretation and asks for a test that is monotone in the individual observed effects. Therefore, a maximum-type test will typically be preferable when testing hypotheses across multiple models.

### 3.4 Multiple testing procedures

The tests considered so far test a global null hypothesis $H_{0}:L\beta =r$. Each row of this equation corresponds to an elementary null hypotheses $H_{i}:(L\beta ){\&}_{i}=r_{i},i=1,\ldots ,c$ while controlling the FWER in the strong sense requires an appropriate multiple testing procedure.

#### 3.4.1 Single step procedure

Based on the maximum-type Wald test, a single step multiple testing procedure with strong FWER control at level *α* rejects *H*_*i*_ if $|((L\overset{\wedge }{\beta }){\&}_{i}-r_{i})/{\overset{\wedge }{\mathrm{SE}}}_{i}|>q_{1-\alpha }$. Corresponding simultaneous 1 – *α* Wald confidence intervals for Lβ are given by

$$\left(L\overset{\wedge }{\beta }){\&}_{i}\pm q_{1-\alpha }{\overset{\wedge }{\mathrm{SE}}}_{i}\;\;(6\right)$$

$q_{1-\alpha }$ may be replaced by $t_{1-\alpha }$ to use a multivariate *t* reference distribution. Note that the single step procedure based on the maximum-type score test may not control the FWER because its multivariate reference distribution is valid only under the global null hypothesis *H*_0_.

#### 3.4.2 Closed testing procedure

A general and more powerful multiple testing procedure can be constructed with the closed testing principle.^27^ Let I={1,…,c} denote the index set of the elementary hypotheses. According to the closed testing principle, an intersection hypothesis $\cap_{i\in S}H_{i},S\subseteq I$ can be rejected with strong control of the FWER at level *α* if all intersection hypotheses $\cap_{i\in S'}H_{i}$ with S⊆S' are rejected by a local level *α* test. Note that in the context of linear hypotheses, the intersection hypothesis $\cap_{i\in S}H_{i}$ corresponds to the set of linear contrasts $(L\beta ){\&}_{i\in S}=(r){\&}_{i\in S}$. Thus, to construct a multiple testing procedure, we define for each such intersection hypothesis a level *α* test given by one of the tests described above and decide on the intersection and elementary hypothesis according to the closed testing principle.

If the matrix L is not of full rank, some intersection hypotheses are equivalent. It is therefore not necessary, and in fact would reduce the power of the procedure, to test all intersection hypotheses in the closed testing procedure. Instead, the test of a hypothesis $\cap_{i\in S}H_{i}$ may be substituted by the test for an equivalent hypothesis $\cap_{i\in S'}H_{i}$ with S⊂S'. Shaffer^28^ describes a general method to identify redundant intersection hypotheses. In the context of linear contrast tests, and under the assumption that Lβ=r has at least one solution, two intersection hypotheses $\cap_{i\in S}H_{i}$ and $\cap_{i\in S'}H_{i}$ are equivalent if the corresponding contrast matrices $(L){\&}_{i\in S}$ and $(L){\&}_{i\in S'}$ define the same region in the parameter space. This is the case if $\text{rank}\left({\left(\text{L}\right)}_{i\in S}\right)=\text{rank}\left({\left({\left(\text{L}\right)}_{i\in S}^{T},{\left(\text{L}\right)}_{i\in S\prime }^{T}\right)}^{T}\right)$.

Multiplicity adjusted p-values for the test of $\cap_{i\in S}H_{i}$ are defined as the smallest family-wise significance level for which $\cap_{i\in S}H_{i}$ can be rejected using the closed testing procedure or, equivalently, as the maximum of local p-values for all local tests of $\cap_{i\in S'}H_{i},S\subseteq S'$.

When using maximum-type tests, a weighted closed testing procedure as discussed by Xi et al.^29^ may be applied to account for differences in importance of the tested hypotheses, depending on the study aims.

## 4 Example – A retina disease study

In a recent exploratory study, the association between two metric endpoints $Y^{\left(1\right)}$ and $Y^{\left(2\right)}$, both measuring retinal function, and three categorical variables $X^{\left(1\right)},X^{\left(2\right)},X^{\left(3\right)}$, each representing the condition of one of three retinal cell layers, was analyzed. $X^{\left(1\right)}$ and $X^{\left(2\right)}$ allow for three stages of deterioration in {0, 1, 2} and $X^{\left(3\right)}$ comprises two stages {0, 1}. Within each eye, the set of variables was measured at 29 to 51 distinct locations defined through a common grid. In total, the study data comprise observations from 1489 locations in 35 eyes of 18 patients. Six marginal analysis models (7) to (12) were defined.

(7) $$E(Y_{\mathrm{ij}}^{\left(1\right)})=\beta_{0}^{\left(1\right)}+1_{\left\{X_{\mathrm{ij}}^{\left(1\right)}=1\right\}}\beta_{1}^{\left(1\right)}+1_{\left\{X_{\mathrm{ij}}^{\left(1\right)}=2\right\}}\beta_{2}^{\left(1\right)}$$

(8) $$E(Y_{\mathrm{ij}}^{\left(1\right)})=\beta_{0}^{\left(2\right)}+1_{\left\{X_{\mathrm{ij}}^{\left(2\right)}=1\right\}}\beta_{1}^{\left(2\right)}+1_{\left\{X_{\mathrm{ij}}^{\left(2\right)}=2\right\}}\beta_{2}^{\left(2\right)}$$

(9) $$E(Y_{\mathrm{ij}}^{\left(1\right)})=\beta_{0}^{\left(3\right)}+1_{\left\{X_{\mathrm{ij}}^{\left(3\right)}=1\right\}}\beta_{1}^{\left(3\right)}$$

(10) $$E(Y_{\mathrm{ij}}^{\left(2\right)})=\beta_{0}^{\left(4\right)}+1_{\left\{X_{\mathrm{ij}}^{\left(1\right)}=1\right\}}\beta_{1}^{\left(4\right)}+1_{\left\{X_{\mathrm{ij}}^{\left(1\right)}=2\right\}}\beta_{2}^{\left(4\right)}$$

(11) $$E(Y_{\mathrm{ij}}^{\left(2\right)})=\beta_{0}^{\left(5\right)}+1_{\left\{X_{\mathrm{ij}}^{\left(2\right)}=1\right\}}\beta_{1}^{\left(5\right)}+1_{\left\{X_{\mathrm{ij}}^{\left(2\right)}=2\right\}}\beta_{2}^{\left(5\right)}$$

(12) $$E(Y_{\mathrm{ij}}^{\left(2\right)})=\beta_{0}^{\left(6\right)}+1_{\left\{X_{\mathrm{ij}}^{\left(3\right)}=1\right\}}\beta_{1}^{\left(6\right)}$$

Here, 1 is the indicator function. Each model was fit using the GEE method with patient as clustering variable and specifying an exchangeable working correlation structure. Note that the robust variance estimation via the GEE approach was preferred over a mixed model since the true correlation structure is most likely too complicated to be explicitly modelled correctly.

Six null hypotheses, addressing the association between an outcome and one independent factor, are regarded in the study: $H_{1}:\beta_{1}^{\left(1\right)}=\beta_{2}^{\left(1\right)}=0,H_{2}:\beta_{1}^{\left(2\right)}=\beta_{2}^{\left(2\right)}=0,H_{3}:\beta_{1}^{\left(3\right)}=0,H_{4}:\beta_{1}^{\left(4\right)}=\beta_{2}^{\left(4\right)}=0,H_{5}:\beta_{1}^{\left(5\right)}=\beta_{2}^{\left(5\right)}=0$ and $H_{6}:\beta_{1}^{\left(6\right)}=0$. We illustrate the application of multivariate inference for the set of hypotheses $\left\{H_{i},i=1,\ldots ,6\right\}$ based on the joint distribution of the coefficients from all six models. Following the discussion at the end of Section 3.3, we use maximum tests. Define the contrast matrices $L^{\left(1\right)}=L^{\left(2\right)}=L^{\left(4\right)}=L^{\left(5\right)}=((0,1,0){\&}^{T},(0,0,1){\&}^{T},(0,1,-1){\&}^{T}){\&}^{T}$ and let $L^{\left(3\right)}=L^{\left(6\right)}=(0,1)$. The right-hand side vector is r=0. Then, the intersection hypotheses $\cap_{i\in S}H_{i},S\subseteq \{1,\ldots ,6\}$ correspond to $L_{S}\beta_{S}=0$ where $L_{S}$ is a block diagonal matrix composed of the matrices $L^{\left(i\right)},i\in S$ and $\beta_{S}$ is the stacked vector of $\beta^{\left(i\right)},i\in S$. Each of these hypotheses is tested by a maximum-type Wald test, using the bias-adjusted covariance matrix estimate and a multivariate *t*-distribution with *df* = *K* – 3 = 18 – 3 = 15 degrees of freedom (or *df* = *K* – 2 = 16 if only *H*_3_ and *H*_6_ are involved) as reference distribution. Adjusted p-values resulting from the closed test for $\left\{H_{i},i=1,\ldots ,6\right\}$ are calculated as described in Section 3.4. For comparison, adjusted p-values according to the Bonferroni–Holm method^30^ are also calculated.

Table 1 shows the unadjusted p-values of the separate maximum tests for $H_{1},\ldots ,H_{6}$, adjusted p-values resulting from the application of the Bonferroni-Holm method to the former unadjusted p-values and adjusted p-values calculated by applying the closed testing procedure outlined in Section 3.4 to the set of hypotheses $\left\{H_{1},\ldots ,H_{6}\right\}$. Hypotheses *H*_1_, *H*_2_ and *H*_3_ are rejected at a family-wise 5% significance level with both multiplicity adjustments. Also, for *H*_4_ and *H*_5_, both methods give similar results and do not reject. The test for *H*_6_ has a local p-value of 0.0237, and the Bonferroni–Holm adjustment results in an adjusted p-value of 0.0710, such that the hypothesis is not rejected with this procedure. In contrast, the closed test based on maximum tests across multiple marginal GEE models results in a multiplicity adjusted p-value of 0.0362, allowing for the rejection of *H*_6_.

**Table 1.** Unadjusted and adjusted p-values for maximum-type Wald tests in the retina disease example.

Hypothesis	Unadjusted p	Holm	mmmGEE
*H*_1_	<0.0001	<0.0001	<0.0001
*H*_2_	<0.0001	<0.0001	<0.0001
*H*_3_	0.0001	0.0002	0.0002
*H*_4_	0.0773	0.0828	0.0819
*H*_5_	0.0414	0.0828	0.0819
*H*_6_	0.0237	0.0710	0.0363

Adjusted p-values are calculated using the Bonferroni-Holm method (Holm) and the closed testing procedure applied to contrasts across multiple marginal generalized estimating equation (GEE) models (mmmGEE).

## 5 Type I error rate and power comparisons in finite samples

A simulation study was performed to investigate the power and type I error rate of the proposed hypothesis tests as well as the coverage probability of simultaneous confidence intervals in settings with multiple differently scaled endpoints. The supplemental material Section S.2 contains a further simulation study assessing the coverage of simultaneous confidence intervals based on multiple marginal GEE models in a recently planned clinical trial in actinic keratosis with a continuous and a binary endpoint.

### 5.1 Data generating model

We considered scenarios with M∈{3,6,9,12} endpoints $Y_{\mathrm{ij}}^{\left(m\right)},m=1,\ldots ,M,$ with subjects indexed i=1,…,K and repeated measurements indexed $j=1,\ldots ,n_{i}$. For each subject, *n*_*i*_ was randomly drawn from a discrete uniform distribution on {2, 3, 4}. In each scenario, one third of the endpoints were continuous $Y_{\mathrm{ij}}^{\left(m\right)}\in \mathbb{R},m=1,\ldots ,M/3,$ with a conditional normal distribution (conditional on the covariates *Group*_*i*_ and $x_{i}^{\left(m\right)}$) with variance 1 and mean

(13) $$\mu_{\mathrm{ij}}^{\left(m\right)}=\beta_{0}^{\left(m\right)}+\mathrm{Grou}p_{i}\beta_{1}^{\left(m\right)}+x_{i}^{\left(m\right)}\beta_{2}^{\left(m\right)}$$

One third were count data endpoints $Y_{\mathrm{ij}}^{\left(m\right)}\in \{0,1,2,\ldots \},m=M/3+1,\ldots ,2M/3,$ with a conditional negative binomial distribution with variance $mu_{ij}^{\left(m\right)}+mu_{ij}^{\left(m\right)}{\;}^{2}$ and mean structure

(14) $$\text{log}\mu_{\mathrm{ij}}^{\left(m\right)}=\beta_{0}^{\left(m\right)}+\mathrm{Grou}p_{i}\beta_{1}^{\left(m\right)}+x_{i}^{\left(m\right)}\beta_{2}^{\left(m\right)}$$

The final third were binary endpoints $Y_{\mathrm{ij}}^{\left(m\right)}\in \{0,1\},m=2M/3+1,\ldots ,M,$ with a conditional Bernoulli distribution with mean structure.

(15) $$\text{log}\frac{\mu_{\mathrm{ij}}^{\left(m\right)}}{1-\mu_{\mathrm{ij}}^{\left(m\right)}}=\beta_{0}^{(m}+\mathrm{Grou}p_{i}\beta_{1}^{\left(m\right)}+x_{i}^{\left(m\right)}\beta_{2}^{\left(m\right)}$$

Here, *Group* is a binary variable in {0, 1}, e.g. indicating treatment versus control. In the simulation study, inference for the corresponding coefficients $\beta_{1}^{\left(m\right)},m=1,\ldots ,M$ was studied. $x_{i}^{\left(m\right)}$ corresponds to a covariate that is specific for the *m*-th outcome and that is observed once for each patient. The vector $\left(x_{i}^{\left(1\right)},\ldots ,x_{i}^{\left(M\right)}\right)$ was drawn from a multivariate normal distribution with zero mean vector, unit variances and all pair-wise correlations set to 0.4.

To simulate correlated observations $\left(Y_{i1}^{\left(1\right)},\ldots ,Y_{in_{i}}^{\left(1\right)},\ldots ,Y_{i1}^{\left(M\right)},\ldots ,Y_{in_{i}}^{\left(M\right)}\right)$, for each subject, i=1,…,K first a latent multivariate normal vector $\xi_{i}=(\xi_{i1}^{\left(1\right)},\ldots ,\xi_{in_{i}}^{\left(1\right)},\ldots ,\xi_{i1}^{\left(M\right)},\ldots ,\xi_{in_{i}}^{\left(M\right)})$ with mean zero and unit variances was sampled. Elements of $\xi_{i}$ corresponding to repeated observations of the same endpoint had pair-wise correlations of 0.75. The correlation between elements corresponding to different endpoints was 0, 0.25, 0.5 or 0.75 to model zero, weak, intermediate and strong correlations between endpoints. The intermediate correlation was our base setting used in most simulation scenarios. Observations on the continuous, count data and binary outcomes were then obtained by the quantile substitution $Y_{\mathrm{ij}}^{\left(m\right)}=Q_{\mathrm{ij}}^{\left(m\right)}(\Phi (\xi_{\mathrm{ij}}^{\left(m\right)}))$. Here, Φ is the standard normal distribution function and $Q_{\mathrm{ij}}^{\left(m\right)}$ is the quantile function of, depending on the type of endpoint, a normal distribution with mean $\mu_{\mathrm{ij}}^{\left(m\right)}$ and variance 1, a negative binomial distribution with mean $\mu_{\mathrm{ij}}^{\left(m\right)}$ and dispersion parameter 1 (resulting in a variance of $\mu_{ij}^{\left(m\right)}+\mu_{ij}^{\left(m\right)}{\;}^{2}$), or a Bernoulli distribution with mean $\mu_{\mathrm{ij}}^{\left(m\right)}$.

The resulting pair-wise correlations between the marginal Wald or score statistics were close to the corresponding correlation between the latent variables or, for pairs involving non-continuous endpoints, slightly below the latent variable correlation.

### 5.2 Hypothesis tests and confidence intervals

We tested the global null hypothesis $H_{0}:\beta_{1}^{\left(1\right)}=\ldots =\beta_{1}^{\left(M\right)}=0$. For the scenario with *M* = 3 endpoints and intermediate correlations, we also tested the elementary hypotheses $H_{1}:\beta_{1}^{\left(1\right)}=0,H_{2}:\beta_{1}^{\left(2\right)}=0$ and $H_{3}:\beta_{1}^{\left(3\right)}=0$, using the closed testing approach of Section 3. The estimates ${\overset{\wedge }{\beta }}^{\left(1\right)},\ldots ,{\overset{\wedge }{\beta }}^{\left(M\right)}$ were calculated from marginal GEE models with mean structures as defined in equations (13) to (15), canonical variance functions for linear, Poisson and logistic regression models, respectively, and subject as clustering variable. Note that inference in the Poisson type GEE models is valid in the presence of overdispersion due to the robust covariance estimation. The exchangeable working correlation structure was specified for all models.

We investigated the performance of the following hypothesis tests as described in Section 3: The quadratic form Wald test using the chi-squared, *F* or scaled *F* reference distribution, the maximum-type Wald test using the multivariate normal or multivariate *t*-distribution as reference distribution, the quadratic form score test with a chi-squared reference distribution and the maximum-type score test with a multivariate normal reference distribution. For the Wald statistics, all tests were calculated, both, with and without bias adjustment of the covariance matrix estimate. For comparison, we further included Bonferroni–Holm tests for the maximum-type Wald statistics using the 1-*α*/2/*M* quantile of a univariate normal or univariate *t*-distribution as critical quantile.

Simultaneous confidence intervals according to equation (6) were calculated for scenarios with three endpoints and intermediate correlations for $\beta_{1}^{\left(1\right)},\beta_{1}^{\left(2\right)}$ and $\beta_{1}^{\left(3\right)}$, with and without bias adjustment of the covariance matrix estimate and based on a critical quantile of either a multivariate normal or *t*-distribution.

For methods based on a multivariate *t*-distribution or an *F*-distribution, *df* = *K* – 3 error degrees of freedom were used. For all tests, the nominal type I error rate was *α* = 0.05.

### 5.3 Simulation scenarios

We considered scenarios with *K* = 40 and *K* = 100 subjects, with *K*/2 subjects in each class of the *Group* variable. The true coefficients in the data generating models were $\beta^{\left(m\right)}=(0,\beta_{1}^{\left(m\right)},0.25)$ for the continuous endpoints, $\beta^{\left(m\right)}=(0,\beta_{1}^{\left(m\right)},0.25)$ for the count data endpoints and $\beta^{\left(m\right)}=(-0.75,\beta_{1}^{\left(m\right)},0.25)$ for the binary endpoints. To investigate type I error rates, simulations were performed under the global null hypothesis where $\beta_{1}^{\left(1\right)}=\ldots =\beta_{1}^{\left(M\right)}=0$. For the case *M* = 3, we studied the power under alternative hypotheses with an effect in (a) all three endpoints, (b) in endpoints 2 and 3 only and (c) in endpoint 3 only. For M∈{6,9,12}, we considered scenarios with an effect in all endpoints. To model an effect in the respective endpoint with *K* = 40 subjects, we set the coefficients $\beta_{1}^{\left(m\right)}$ to 0.75, 0.95 and 1.5 for continuous, count and binary endpoints, respectively. In scenarios with *K* = 100 subjects, the respective values for $\beta_{1}^{\left(m\right)}$ were 0.45, 0.6 and 0.9. This choice of parameters results in similar expectations of the marginal Wald and score statistics close to 2.5 for each endpoint under the respective marginal alternative in all scenarios. For an unadjusted single-endpoint test based on a standard normal distribution, this corresponds to a power of approximately 70%.

For each scenario, 10^5^ simulation runs were performed, except for more computation intensive simulations addressing the effect of increasing numbers of endpoints, where 2 × 10^4^ simulation runs were performed. The power for each test was calculated as the proportion of simulation runs in which *H*_0_ was rejected.

### 5.4 Simulation results

Simulation results regarding the type I error rate of tests for *H*_0_ with *M* = 3 and *M* = 12 and intermediate correlations are shown in Table 2. Using any of the Wald tests without small sample adjustments leads to severe inflation of the type I error rate, and the inflation is increasing with the number of endpoints and decreasing with the number of subjects. Among the studied scenarios, the type I error rate was up to 10% for the unadjusted maximum-type Wald test and up to 41% for the unadjusted quadratic form Wald test. Both, the bias adjustment of the covariance estimate and the distributional approximation via an *F*- or a multivariate *t*-distribution, are required to control the type I error rate at the nominal level. For the quadratic form Wald test, using a scaled *F* statistic in analogy to Hotelling's *T*^2^ test is required to control the type I error rate across all scenarios.

**Table 2.** Type I error rate in the simulations with *M* = 3 endpoints and *M* = 12 endpoints with intermediate correlations when testing $H_{0}:\beta_{1}^{\left(1\right)}=\ldots =\beta_{1}^{\left(M\right)}=0$ using the methods described in Section 3 or a Bonferroni test.

Statistic	Type	Approximation	Bias adj.	Type I error rate (%)			
				*M* = 3		*M* = 12	
				*K* = 40	*K* = 100	*K* = 40	*K* = 100
Wald	Quadratic	Chi-squared	no	9.8	6.8	41.0	15.1
			yes	6.2	5.5	28.1	11.6
		F	no	7.6	6.1	30.0	12.1
			yes	4.7	4.9	19.1	9.0
		Scaled F	no	6.4	5.6	8.6	6.7
			yes	3.8	4.5	4.3	4.7
	Maximum	MVN	no	8.1	6.1	10.4	6.8
			yes	5.2	5.1	6.2	5.5
		MVT	no	6.5	5.6	7.6	6.0
			yes	4.0	4.6	4.3	4.7
		Normal-Bonferroni	no	7.3	5.5	8.4	5.3
			yes	4.7	4.5	4.9	4.2
		t-Bonferroni	no	5.8	5.0	5.6	4.5
			yes	3.5	4.0	3.0	3.5
Score	Quadratic	Chi-squared	no	4.6	4.9	2.2	4.0
	Maximum	MVN	no	5.0	5.0	4.6	4.9

The tests are based on quadratic form or maximum-type Wald or score statistics. The reference distributions are chi-squared, *F*, scaled *F*, MVN or MVT. All Wald tests were performed with and without bias adjustment (column 'Bias adj.'). The considered sample sizes were *K* = 40 and *K* = 100. The results are based on 10^5^ simulation runs. MVN: multivariate normal; MVT: multivariate *t*.

The score statistics exhibit favorable properties in the simulation, with type I error rates very close to the nominal level. No small sample adjustment in terms of bias adjustment or refined distributional approximation is required.

We studied the power of those procedures for which type I error rate control was observed in the simulation. For *M* = 3 endpoints and intermediate correlations, the results for the test of $H_{0}:\beta_{1}^{\left(1\right)}=\beta_{1}^{\left(2\right)}=\beta_{1}^{\left(3\right)}=0$ under scenarios (a), (b) and (c) with effects in three, two and one endpoint are shown in Table 3. Throughout these scenarios, the multivariate maximum-type Wald test has a power advantage of some percentage points over the Bonferroni test. Furthermore, the score test is considerably more powerful than the Wald test and this holds for, both, the quadratic form statistics and the maximum statistics.

**Table 3.** Power to reject $H_{0}:\beta_{1}^{\left(1\right)}=\beta_{1}^{\left(2\right)}=\beta_{1}^{\left(3\right)}=0$ with selected testing approaches that control the type I error rate (see Table 2 for details).

Statistic	Type	Approx.	Bias adj.	Power (%)					
				K = 40			K = 100		
				a	b	c	a	b	c
Wald	Quadratic	Scaled F	yes	67.5	83.7	63.1	70.5	86.8	70.5
Wald	Maximum	MVT	yes	77.7	71.2	53.7	78.8	72.9	57.8
Wald	Maximum	t-Bonferroni	yes	75.5	68.6	50.8	76.9	70.9	55.5
Score	Quadratic	Chi-squared	no	72.1	86.7	74.5	71.9	87.7	74.2
Score	Maximum	MVN	no	80.7	74.3	64.7	79.7	73.9	61.6

The power was calculated for sample sizes *K* = 40 and *K* = 100 in three different scenarios. In scenario (a), there was an effect in all three endpoints ($\beta_{1}^{\left(i\right)}\neq 0,i=1,2,3$), in scenario (b) there was an effect in endpoints 2 and 3 ($\beta_{1}^{\left(1\right)}=0$), and in scenario (c) there was an effect in endpoint 3 only ($\beta_{1}^{\left(1\right)}=\beta_{1}^{\left(2\right)}=0$). See text for the exact values of non-zero coefficients. The results are based on 10^5^ simulation runs. MVN: multivariate normal; MVT: multivariate *t*.

The quadratic form Wald test has more power to reject the global null hypothesis in scenario (b) and almost identical power in scenario (c) compared to scenario (a). This observation is in agreement with the discussion in Section 3.3. Under the simulation settings, the correlation matrix of $\left({\overset{\wedge }{\beta }}_{1}^{\left(1\right)},{\overset{\wedge }{\beta }}_{1}^{\left(2\right)},{\overset{\wedge }{\beta }}_{1}^{\left(3\right)}\right)$ is approximately $C=((1,0.5,0.5){\&}^{T},(0.5,1,0.5){\&}^{T},(0.5,0.5,1{)}^{T})$. Thus, the non-centrality parameter of the quadratic form Wald test is approximately $(2.5,2.5,2.5)C^{-1}(2.5,2.5,2.5){\&}^{T}=9.4$ under scenario (a), $(0,2.5,2.5)C^{-1}(0,2.5,2.5){\&}^{T}=12.5$ under scenario (b) and $(0,0,2.5)C^{-1}(0,0,2.5){\&}^{T}=9.4$ under scenario (c). This corresponds to a theoretical power of 73%, 86% and 73%, respectively, under a chi-squared approximation, which is in line with the simulation results in Table 3. For the quadratic form score test, similar results hold. Under the simulation settings the pair-wise correlations between the marginal score statistics were approximately 0.5, however the expectation of the score statistics were slightly different, with values approximately 2.4, 2.3 and 2.6 for the continuous, the count data and the binary endpoint, respectively. Hence, even scenario (c), in which the only effect is on the binary endpoint, results in more power than scenario (a) for the quadratic form score test.

We further studied for scenario (a), the power of closed testing procedures, which utilize the above tests for each intersection hypothesis, to reject particularly *H*_1_, *H*_2_ or *H*_3_, as well as the power to reject at least one elementary hypothesis or all elementary hypotheses, see Table 4. For comparison, the closed testing procedure based on Bonferroni tests (which results in the Bonferroni-Holm procedure) is included. Similar to the results on the global test, the maximum-type Wald test is more powerful than the Bonferroni–Holm test and the score tests are for most decisions more powerful than the Wald tests.

**Table 4.** Power of closed testing procedures to reject $H_{1}:\beta_{1}^{\left(1\right)}=0,H_{2}:\beta_{1}^{\left(2\right)}=0,H_{3}:\beta_{1}^{\left(3\right)}=0$, at least one elementary hypothesis (any *H_i_*), or all elementary hypotheses.

K	Statistic	Type	Approx.	Bias adj.	*H*_1_	*H*_2_	*H*_3_	any *H*_*i*_	all *H*_*i*_
40	Wald	Quadratic	scaled F	yes	53.1	54.0	54.3	67.1	41.4
40	Wald	Maximum	MVT	yes	59.0	59.4	60.6	77.7	42.2
40	Wald	Maximum	t-Bonferroni	yes	57.4	57.9	58.9	75.5	41.5
40	Score	Quadratic	Chi-squared	no	57.7	53.8	64.0	71.7	46.0
40	Score	Maximum	MVN	no	63.0	58.1	69.2	80.7	46.6
100	Wald	Quadratic	Scaled F	yes	54.3	56.3	57.8	70.1	43.1
100	Wald	Maximum	MVT	yes	58.8	60.8	62.8	78.8	43.2
100	Wald	Maximum	t-Bonferroni	yes	57.5	59.4	61.4	76.9	42.7
100	Score	Quadratic	Chi-squared	no	56.3	56.1	61.5	71.5	45.0
100	Score	Maximum	MVN	no	60.6	60.2	66.1	79.7	45.1

Results are shown for the simulation scenario (a) with an effect in all endpoints. The results are based on 10^5^ simulation runs. MVN: multivariate normal; MVT: multivariate *t*.

The coverage probability of simultaneous confidence intervals for $\left(\beta_{1}^{\left(1\right)},\beta_{1}^{\left(2\right)},\beta_{1}^{\left(3\right)}\right)$ did not depend on the actual values of the coefficients, up to simulation error. The observed values for scenario (a) are shown in Table 5. Both considered small sample adjustments are required to achieve a coverage probability of at least the nominal value of 95%. Similar results were observed in the simulation contained in the supplemental material Section S.2.

**Table 5.** Simultaneous coverage probability of nominal 95% simultaneous confidence intervals for $\left(\beta_{1}^{\left(1\right)},\beta_{1}^{\left(2\right)},\beta_{1}^{\left(3\right)}\right)$ for scenario (a) with sample sizes *K* = 40 and *K* = 100.

Approximation	Bias adj.	*K* = 40	*K* = 100
MVN	no	91.9	93.7
MVN	yes	94.7	94.8
MVT	no	93.4	94.3
MVT	yes	95.8	95.2

The intervals were calculated based on an approximating MVN or MVT distribution, with and without bias adjustment for the covariance matrix estimate. The results are based on 10^5^ simulation runs. MVN: multivariate normal; MVT: multivariate *t*.

**Table:** 

library(mmmgee) data(datasim) mod1<-geem2(Y.lin∼gr.lang+x1,id=id,data=datasim, family="gaussian",corstr="exchangeable") mod2<-geem2(Y.poi∼gr.lang+x2,id=id,data=datasim, family="poisson",corstr="exchangeable") mod3<-geem2(Y.bin∼gr.lang+x3,id=id,data=datasim, family="binomial",corstr="exchangeable") Li<-matrix(c(0,1,0),nrow=1) mmmgee.test(list(mod1,mod2,mod3),L=list(Li,Li,Li), statistic="Wald",type="maximum",biascorr=TRUE, asymptotic=FALSE,closed.test=TRUE)

**Table:** 

Hypothesis tests for linear contrasts in multiple marginal GEE models Statistic: Maximum-type Wald statistic Approximation: Multivariate t Alternative: Undirected Global test: Max|T| = 3.368, df = 37, p-value = 0.005021 Closed testing procedure: contrast estimate rhs p.unadj p.adjusted 1 1 0.752 0 0.004586 0.009038 2 2 1.309 0 0.006406 0.009038 3 3 1.931 0 0.001778 0.005021

**Figure 1.**
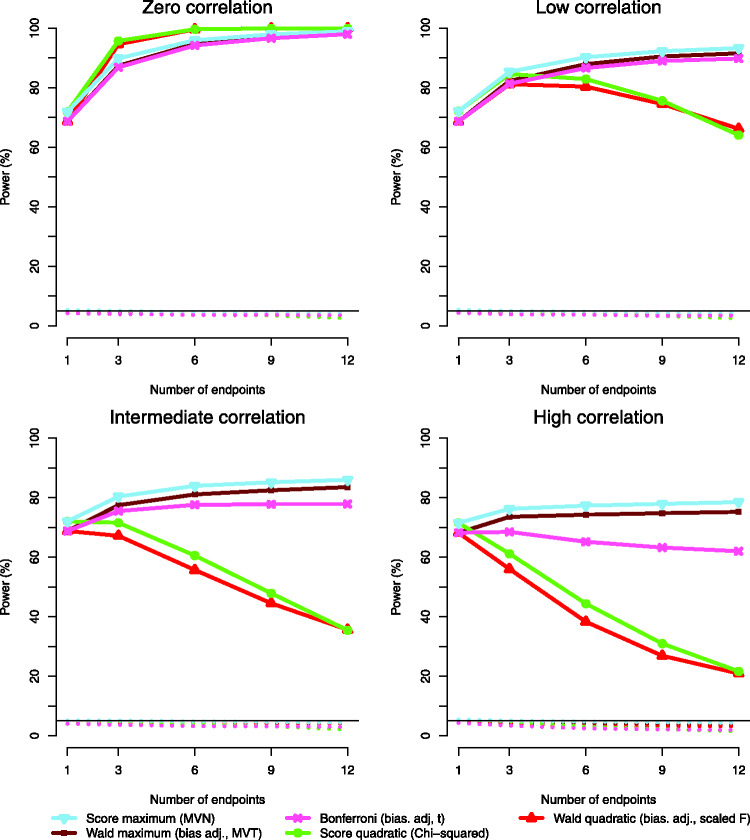
Power to reject the global null hypothesis $H_{0}:\beta_{1}^{\left(1\right)}=\ldots =\beta_{1}^{\left(M\right)}=0$ under an alternative with an effect in all *M* endpoints (solid lines) and type I error rate under *H*_0_ (dotted lines) for scenarios with *K* = 40 subjects and increasing number of endpoints. The correlation between marginal Wald or score statistics is approximately 0.25, 0.5 and 0.75 in the scenarios with low correlation, intermediate correlation and high correlation. The nominal level of 0.05 is indicated by a horizontal line. The studied tests are listed in the legend. The information in parentheses shows that the bias adjustment for the covariance matrix was applied to all tests using Wald statistics; furthermore, the reference distributions with abbreviations as in Table 2 are indicated. The results are based on 2 × 10^4^ simulation runs.

In a further simulation study, we investigated the impact of increasing the number of endpoints and increasing the correlation between endpoints for scenarios with *K* = 40 subjects and an effect in all endpoints under the alternative. We included only those tests that controlled the type I error rate. The simulation results are shown in Figure 1. Here, we also included the case *M* = 1. To allow for an unambiguous comparison with the case of multiple endpoints of different types, we computed for *M* = 1 the results for models with a single continuous, count data and binary endpoint, respectively, and plotted the average power across these three models from a total of 2 × 10^4^ simulations.

The benefit of the maximum-type Wald and score tests over the Bonferroni test becomes more pronounced for larger correlations and their power (under the considered alternative with an effect in all endpoints) is increasing with the number of endpoints. Under high correlations, the power of the maximum-type Wald and score test is approximately constant with an increasing number of endpoints, whereas the power of the Bonferroni test is decreasing. Note that in an extreme case of correlation 1, the maximum test would be identical to a test for a single endpoint, with no loss in power, whereas the Bonferroni test would correspond to a single-endpoint test at level *α*/*M* hence loosing power.

The power of the quadratic form Wald and score tests depends strongly on the correlation between endpoints. As seen in Table 3 and discussed in Section 3.3, if there is an effect in all endpoints and the endpoints are positively correlated, the direction of the effects is not the direction of deviations from the null hypothesis which are considered particularly large by the metric of these tests. This becomes more pronounced with an increasing number of endpoints and increasing correlation, and the power of the quadratic form tests decreases rapidly.

## 6 Software implementation

The proposed test procedures were implemented in the R-package ‘mmmgee’^31^ that is available from the CRAN repository.^32^ The model fitting routines are based on those of the R-package ‘geeM’,^33^ and multivariate normal or *t* distribution probabilities are calculated using the package ‘mvtnorm’.^34^

The mmmgee package provides three main functions: geem2 fits marginal GEE models as described in Sections 2.1 and 2.2. mmmgee calculates the estimate (equation (3)) or the bias adjusted estimate (equation (4)) of the covariance matrix of a stacked vector of regression coefficients from multiple marginal GEE models fitted with geem2. mmmgee.test calculates the multiple hypothesis tests and simultaneous confidence intervals described in Section 3. The latter functions are applied to a list of models fitted with geem2. As a special case, the package may also be applied to test hypotheses within a single GEE model.

An instance of a simulated data set with *M* = 3, *K* = 40 and intermediate correlations as described in Section 5 is included in the package as exemplary data. The R code below invokes an analysis as used in the simulation studies in Section 5. Marginal GEE models are fit for the three endpoints using geem2. The function mmmgee.test is applied to test the global null hypothesis $H_{0}:\beta_{1}^{\left(1\right)}=\beta_{1}^{\left(2\right)}=\beta_{1}^{\left(3\right)}=0$ as well as the elementary hypotheses $H_{1}:\beta_{1}^{\left(1\right)}=0,H_{2}:\beta_{1}^{\left(2\right)}=0$ and $H_{3}:\beta_{1}^{\left(3\right)}=0$ in a closed testing procedure. In the example, a maximum-type Wald test using the bias adjusted covariance matrix estimate and a multivariate *t* reference distribution is requested.

The output includes the test statistic, degrees of freedom and p-value for the test of the global null hypothesis as described in Section 3.1. It further shows the estimated contrasts, which in this case correspond to ${\overset{\wedge }{\beta }}_{1}^{\left(1\right)},{\overset{\wedge }{\beta }}_{1}^{\left(2\right)}$ and ${\overset{\wedge }{\beta }}_{1}^{\left(3\right)}$, the right hand side value of each contrast under the respective null hypothesis, the unadjusted p-values and multiplicity adjusted p-values according to the closed testing procedure of Section 3.4:

See the supplemental material S.3 for further examples.

## 7 Discussion

We proposed a general inference framework for multiple or multivariate outcomes. In particular, we considered the problem of testing multiple hypotheses, the need to account for dependencies of observations within the same subject, the lack of accuracy of asymptotic methods in small sample sizes, and we tried to give some advice on the choice between particular test statistics in the multivariate setting.

The approach is based on multiple marginal models^2^ and requires a distributional model for the joint vector of parameter estimates from these models, but it is not required to assume a fully specified joint model of all outcomes. Marginal GEE models, accounting for dependent observations with respect to one endpoint, fit naturally in the multiple marginal model framework, since both concepts are based on estimating equations that are sums of independent contributions of different subjects and both utilize robust sandwich variance estimation. Note that usual generalized linear models and linear models may be seen as special cases of GEE models and can be included in the proposed framework. As alternative to using multiple marginal models, the correlation between endpoints could be utilized in a weighted estimation of regression coefficients, which may reduce their variance. This idea was studied by Fitzmaurice and Laird^15^ and Rochon^3^ for the special case of a continuous and a binary endpoint. However, as a simulation study by Teixeira-Pinto and Normand^35^ suggests, the improvement is small, and the required additional nuisance parameters may introduce further variability in the case of small samples. We therefore focussed on estimation via marginal models.

The proposed method provides multiplicity adjustment when testing multiple hypotheses or when constructing simultaneous confidence intervals and it takes the correlation between the studied parameters into account. Hence, it is more efficient than commonly used methods that are based on the Bonferroni inequality. Furthermore, while the Bonferroni adjustment is applicable to maximum-type tests, the multivariate normal approximation of the parameter estimates allows for construction of more general test statistics.

Insufficient small sample accuracy of asymptotic methods is a frequent problem that particularly affects studies at early stages of research and studies in rare diseases.^36^ For many asymptotic methods, a major improvement in terms of type I error rate control can be achieved by replacing normal with *t*-distributions and chi-squared with *F*-distributions. In general, the joint distribution of the $(L\overset{\wedge }{\beta }){\&}_{i}/{\overset{\wedge }{\mathrm{SE}}}_{i},i=1,\ldots ,c$ will not be exactly a multivariate *t*-distribution (see the book by Kotz and Nadarajah^21^ for the definition of the multivariate *t*-distribution). Nonetheless, the multivariate *t*-distribution often provides a good approximation.^7,8,22^ Also, the simulation results (see Section 5) suggest that the tests and confidence intervals based on $t_{1-\alpha }$ often have error rates close to the nominal level even for small sample sizes. In any case, the liberalism of the hypothesis tests and confidence intervals is reduced compared to the multivariate normal approximation, because $t_{1-\alpha }>z_{1-\alpha }$. Asymptotically, both approaches are identical.

However, with the exception of certain models under the assumption of normally distributed data, there is no direct way to determine the respective number of error degrees of freedom. We used a simple method essentially subtracting the number of parameters in a model from the number of subjects, with convincing results in the numeric simulations. Several other approaches have been proposed in different contexts and it may be worthwhile to include these in further research.

Pan and Wall^9^ report good results for single GEE models using a Satterthwaite approximation. However, this method requires the estimation of the variance of the covariance matrix estimate. Alternatively, degrees of freedom may be calculated from the effective sample size, which is the number of independent observations that would result in the same efficiency as the observed sample of partially dependent observations.^22^ This method requires that the covariance structure is correctly specified, which is otherwise not required for GEE models. In some cases, it may be reasonable to attribute different degrees of freedom to different tested contrast, e.g. if the number of parameters strongly differs between the marginal models. In that case, a method described by Hasler and Hothorn^8^ may be utilized: For each tested contrast, a critical value is calculated from a multivariate *t*-distribution with common correlation matrix and contrast-specific degrees of freedom. The test decision is based on comparison of the individual test statistics with the respective critical values. Another way to improve the distributional normal approximation of a statistic is to directly calculate and adjust for the error of approximation. Kauermann and Carroll^37^ propose this solution for the case of univariate contrast tests in GEE models. The method could in principle be extended to multivariate tests.

The other small sample improvement we investigated is the bias adjustment of the covariance matrix estimate. We focused on the method of Mancl and DeRouen^7^; however, related methods proposed in the literature for single GEE models^14^ may as well be extendable to the multiple marginal models approach.

We regarded the score test as an approximation to the Wald test. Note that generalized score tests with quadratic form test statistics may also be constructed from the score vector U and a generalized inverse of its asymptotic covariance matrix (which may be singular).^26^ For the case of a linear hypothesis *H*_0_, the resulting test is identical to the quadratic form score test motivated via approximation of the Wald test. The latter approach is, however, easily applicable to construct maximum type tests. In the numeric investigations, the score tests did not require adjustments to the asymptotic approximation to control the type I error rate and they were more powerful than the corresponding Wald tests. In contrast to Guo et al.,^38^ who studied quadratic form score tests for single GEE models, we did not observe a conservative behaviour, but the type I error rate was controlled almost exactly at the nominal level. These results may not hold for all possible analysis scenarios but they suggest the score tests as viable small sample alternative to the Wald tests. Confidence sets for Lβ corresponding to a score test may in principle be found as set of all vectors $r'\in {\mathbb{R}}^{c}$ such that $H_{0}:L\beta -r'=0$ is rejected.^38^ To provide contiguous intervals or sets, the test statistic needs to be a convex function of r. In contrast to the Wald statistic, the score statistic depends on r in a non-trivial way, as the statistic is based on a model fitted under the constraint Lβ-r=0. Thus, the required convexity property needs to be checked for each given class of models, which may not be easily done except for some special cases.

We focused on two-sided inference. The extension to one-sided tests for null hypotheses of the form H0'=∩i=1c{(Lβ)&i≤ri} and according one-sided confidence intervals is straight forward for maximum-type Wald tests (compare with Hothorn^20^). The least favorable configuration under H0' is Lβ=r since $\overset{\wedge }{\Sigma }$ is estimated without restriction and the multivariate normal or *t* reference distribution is monotone in the assumed mean vector. Hence, evaluation of the one-sided Wald test under the configuration Lβ=r is sufficient. To extend the maximum-type score test to one-sided hypotheses, the restricted estimate $\overset{∼}{\beta }$ has to be calculated under the according inequality restriction which is subject of further research.

## Supplemental Material

Supplemental material for Simultaneous inference for multiple marginal generalized estimating equation models Click here for additional data file. Supplemental Material for Simultaneous inference for multiple marginal generalized estimating equation models by Robin Ristl, Ludwig Hothorn, Christian Ritz and Martin Posch in Statistical Methods in Medical Research
