# Supplementary material for: Simultaneous inference for multiple marginal generalized estimating equation models
Source: Stat Methods Med Res. 2019 Sep 17;29(6):1746–62. doi: 10.1177/0962280219873005 (PMC7270726; doi:10.1177/0962280219873005)
Supplement: Supplemental material for Simultaneous inference for multiple marginal generalized estimating equation models [file Supplemental_Material.pdf]

Supplemental material for "Simultaneous inference for multiple marginal GEE models"

S.1 Bias-adjusted covariance matrix estimator

The core of the sandwich variance estimator $\hat{\boldsymbol{\Sigma}}$ is the expression $\boldsymbol{U}_i\boldsymbol{U}_i^T = \boldsymbol{D}_i^T\boldsymbol{V}_i^{-1}\boldsymbol{S}_i\boldsymbol{S}_i^T\boldsymbol{V}_i^{-1}\boldsymbol{D}_i$, evaluated at the value of $\hat{\boldsymbol{\beta}}$. Here the estimate $v\hat{a}r(\boldsymbol{Y}_i) = \boldsymbol{S}_i\boldsymbol{S}_i^T$ is calculated from the residuals $\boldsymbol{S}_i = \boldsymbol{Y}_i - \hat{\boldsymbol{\mu}}_i$. In the setting of a single GEE model, Mancl and DeRouen [1] used the first order expansion $\boldsymbol{S}_i^{(m)} \approx \boldsymbol{e}_i^{(m)} + \frac{\partial \boldsymbol{e}_i^{(m)}}{\partial \boldsymbol{\beta}^{(m)}}(\hat{\boldsymbol{\beta}}^{(m)} - \boldsymbol{\beta}^{(m)})$, with $\boldsymbol{e}_i^{(m)} = \boldsymbol{Y}_i^{(m)} - \boldsymbol{\mu}_i^{(m)}$ to derive an approximation of the bias of $\boldsymbol{S}_i^{(m)}{\boldsymbol{S}_i^{(m)}}^T$. They showed that $E(\boldsymbol{S}_i^{(m)}{\boldsymbol{S}_i^{(m)}}^T) \approx (\boldsymbol{I}_i^{(m)} - \tilde{\boldsymbol{H}}_{ii}^{(m)})var(\boldsymbol{Y}_i^{(m)})(\boldsymbol{I}_i^{(m)} - \tilde{\boldsymbol{H}}_{ii}^{(m)})$ where $\tilde{\boldsymbol{H}}_{ii}^{(m)} = \boldsymbol{D}_i^{(m)}{\boldsymbol{H}^{(m)}}^{-1}{\boldsymbol{D}_i^{(m)}}^T{\boldsymbol{V}_i^{(m)}}^{-1}$, and $\boldsymbol{I}_i^{(m)}$ denotes the identity matrix of same dimension. They proposed a bias-adjusted sandwich estimator in which $v\hat{a}r(\boldsymbol{Y}_i^{(m)}) = \boldsymbol{S}_i^{(m)}{\boldsymbol{S}_i^{(m)}}^T$ is replaced by $v\hat{a}r_{adj}(\boldsymbol{Y}_i^{(m)}) = (\boldsymbol{I}_i^{(m)} - \tilde{\boldsymbol{H}}_{ii}^{(m)})^{-1}\boldsymbol{S}_i^{(m)}{\boldsymbol{S}_i^{(m)}}^T(\boldsymbol{I}_i^{(m)} - \tilde{\boldsymbol{H}}_{ii}^{(m)})^{-1}$. The arguments of Mancl and DeRouen similarly apply to the stacked vectors $\boldsymbol{U}_i$, $\boldsymbol{Y}_i$ and $\boldsymbol{S}_i$ from multiple models, such that $E(\boldsymbol{S}_i\boldsymbol{S}_i^T) \approx (\boldsymbol{I}_i - \tilde{\boldsymbol{H}}_{ii})var(\boldsymbol{Y}_i)(\boldsymbol{I}_i - \tilde{\boldsymbol{H}}_{ii})$ with $\tilde{\boldsymbol{H}}_{ii} = \boldsymbol{D}_i\boldsymbol{H}^{-1}\boldsymbol{D}_i^T\boldsymbol{V}_i^{-1}$ and $\boldsymbol{I}_i$ the matching identity matrix. The accordingly adjusted core of the sandwich variance estimator for multiple marginal GEE models is $\hat{\boldsymbol{B}}_{adj} = \sum_{i=1}^K \boldsymbol{D}_i^T\boldsymbol{V}_i^{-1}(\boldsymbol{I}_i - \tilde{\boldsymbol{H}}_{ii})^{-1}\boldsymbol{S}_i\boldsymbol{S}_i^T(\boldsymbol{I}_i - \tilde{\boldsymbol{H}}_{ii})^{-1}\boldsymbol{V}_i^{-1}\boldsymbol{D}_i$ and the bias-adjusted covariance matrix estimator is $\hat{\boldsymbol{\Sigma}}_{adj} = \hat{\boldsymbol{H}}^{-1}\hat{\boldsymbol{B}}_{adj}\hat{\boldsymbol{H}}^{-1}$.

To derive the bias-adjusted covariance estimator for $\tilde{\boldsymbol{B}}$, which may be used in the score test, note that $\tilde{\boldsymbol{\beta}} - \boldsymbol{\beta} \approx \boldsymbol{H}^{-1}(\boldsymbol{L}^T\boldsymbol{\lambda} - \boldsymbol{U}(\boldsymbol{\beta})) = -\boldsymbol{H}^{-1}(\boldsymbol{I} - \boldsymbol{L}^T(\boldsymbol{L}\boldsymbol{H}^{-1}\boldsymbol{L}^T)^{-1}\boldsymbol{L})\boldsymbol{U}(\boldsymbol{\beta})$, where $\boldsymbol{\lambda} = (\boldsymbol{L}\boldsymbol{H}^{-1}\boldsymbol{L}^T)^{-1}\boldsymbol{L}\boldsymbol{H}^{-1}\boldsymbol{U}(\boldsymbol{\beta})$. Use this approximation instead of $\hat{\boldsymbol{\beta}} - \boldsymbol{\beta} \approx -\boldsymbol{H}^{-1}\boldsymbol{U}(\boldsymbol{\beta})$ in the derivation corresponding to Mancl and DeRouen [1].

S.2 A simulation study based on a trial in actinic keratosis

In a recently planned randomized controlled cross-over trial (EudraCT Number 2015-002245-66), a standard photodynamic treatment A for actinic keratosis is compared to three experimental treatment regimens B, C and D with differently reduced radiation doses or fluencies. Each patient receives each treatment in a different skin patch and each patch

is assumed to contain four lesions. The primary study endpoint is the proportion of cleared lesions, which will be analysed in terms of a binary logistic regression GEE model. The main secondary endpoint is pain, experienced during the radiation treatment, measured on a visual analogue scale and analysed by a linear GEE model. We are interested in simultaneous confidence intervals comprising the differences in the proportion of cleared lesions as well as the differences in mean pain scores between the reference treatment and each of the three experimental treatments. The study is ongoing and we here investigate the coverage probability of simultaneous confidence intervals according to equation (2) of the main manuscript under the design assumptions of the actinic keratosis trial.

Simulation set-up

We considered sample sizes $K = 30$, $K = 60$, which is the planned sample size of the actual trial after drop outs, and $K = 100$. For each setting 10^5 data sets were generated. We assumed lesion clearance proportions of 0.7, 0.7, 0.6 and 0.4, in the reference treatment A and the experimental treatments B, C, and D, respectively, and normally distributed pain scores with mean values 5.5, 4.5, 4.5 and 3.5 and a common variance of 1. The within-patient correlation was modelled by sampling a 20 dimensional latent multivariate normal variable for each patient, with 16 dimensions corresponding to clearance of the lesions and four dimensions directly resembling the pain scores under the four treatment regimens. Binary lesion clearance resulted from dichotomization of the according latent variables. Pain scores of different patches had a correlation of 0.6. The latent variables for clearance had a correlation of 0.7 within the same patch and 0.5 between patches. The latent variables for clearance and pain had a correlation of 0.25 within the same patch and were uncorrelated between patches.

Simultaneous confidence intervals

To analyse the data using the multiple marginal model approach, a logistic GEE model

$$\log \frac{\mu_{ij}^{(1)}}{1 - \mu_{ij}^{(1)}} = \beta_0^{(1)} + \mathbb{1}_{\{trt_{ij}=B\}}\beta_1^{(1)} + \mathbb{1}_{\{trt_{ij}=C\}}\beta_2^{(1)} + \mathbb{1}_{\{trt_{ij}=D\}}\beta_3^{(1)}$$

was fit for the binary endpoint clearance and a linear GEE model

$$\mu_{ij}^{(2)} = \beta_0^{(2)} + \mathbb{1}_{\{trt_{ij}=B\}}\beta_1^{(2)} + \mathbb{1}_{\{trt_{ij}=C\}}\beta_2^{(2)} + \mathbb{1}_{\{trt_{ij}=D\}}\beta_3^{(2)}$$

was fit for the metric endpoint pain. In both models, patient was considered as clustering variable and the independence working correlation was specified. The joint covariance matrix of model coefficients was estimated with and without bias adjustment.

Simultaneous nominal 95% Wald confidence intervals according to equation (2) in the main manuscript were calculated for the six coefficients $\beta_i^{(m)}$, $i = 1, 2, 3$, $m = 1, 2$, which correspond to the differences in log-odds of lesion clearence and mean pain score between the alternative treatment regimens and the reference treatment regimen. However, the interpretation of the study results is planned for the difference in proportions not the difference in log-odds. Therefore an estimate for the joint covariance matrix for the differences in lesion clearance proportions and the mean pain differences was derived by applying the delta method (see e.g. chapter 3 in [2]), based on the estimates for the model coefficients and their joint covariance matrix estimate.

Simultaneous nominal 95% Wald confidence intervals were then calculated for the differences in proportion of lesion clearance and the differences of mean pain scores between alternative therapy regimens and the standard regimen. Details are found in the supplementary material.

For both parametrizations, the confidence intervals were calculated based on the joint covariance matrix of model coefficients with or without bias adjustment and utilizing a critical quantile either from a multivariate normal distribution or a multivariate t-distribution with $K - 4$ degrees of freedom.

Simulation results

Table S.1 shows the respective coverage probabilites obtained in the simulation. The results are highly similar for both parametrizations, even though the delta method involves an additional approximation. Without bias adjustment of the covariance matrix estimate and using the multivariate normal distribution, the coverage probability is below the nominal value, however the undercoverage decreases with increasing sample size. Utilizing either the bias adjustment or the multivariate t-distribution improves the coverage probability, though it remains below the nominal value in all scenarios. With both small sample adjustments the observed coverage is closely above the nominal value and this approach is recommended for the actual analysis.

Table S.1: Simultaneous coverage probability (%) of simultaneous confidence intervals for the six contrasts studied in the actinic keratosis trial. Lesion clearance efficacy is either measured in log-odds or proportions of lesion clearance. In the latter case, the delta method is applied in conjunction with the proposed methods to calculate confidence intervals. Confidence intervals are based on the multivariate normal (MVN) or multivariate t-distribution with $K-4$ degress of freedom (MVT) and are calculated with and without bias adjustment of the covariance matrix estimate (column 'Bias adj.'). The considered sample sizes were K = 30, 60 and 100. The nominal coverage probability is 95%. The results are based on 10^5 simulation runs.

		Log-odds and means			Proportions and means		
Approximation	Bias adj.	$K = 30$	$K = 60$	$K = 100$	$K = 30$	$K = 60$	$K = 100$
MVN	no	91.8	93.6	94.2	91.4	93.4	94.1
MVN	yes	93.2	94.2	94.5	92.9	94.0	94.5
MVT	no	94.4	94.7	94.8	94.2	94.5	94.8
MVT	yes	95.5	95.2	95.1	95.3	95.0	95.1

S.3 Exemplary analysis using R

R package

The methods described in the main manuscript were implemented in the R package 'mmmgee' [3] that is available from the CRAN repository. The package can be installed and loaded in R using the commands

```
install.packages("mmmgee")
library(mmmgee)
```

To learn about the included functions for fitting GEE models, estimating the joint covariance matrix and perform hypothesis or calculate confidence intervals use

```
help(geem2)
help(mmmgee)
help(mmmgee.test)
```

Data set

An instance of the simulated data for the actinic keratosis example with $K = 60$ subjects is included in the R package in the data set "keratosis". The data set can be loaded in R by the command

```
data(keratosis)
```

after loading the package. Each line of the data set corresponds to a single lesion. Lesions are numbered consecutively within each treated patch in the variable "lesion". The treatment regimen applied to the respective patch is encoded in the variable "trt" and the patient identity is encoded in the variable "id". A value of 1 in the binary variable "clearance" corresponds to successful lesion clearance. The pain score, found in the variable "pain", is reported for each treatment application and is therefore identical for all four lesions within a treated patch.

Confidence intervals for log-odds and mean differences

With the following R-code, the marginal GEE models are fit, a bias-adjusted estimate for the joint covaraince matrix of the regression coefficients is calculated and simultaneous confidence intervals based on a multivariate t-distribution with $K - 4 = 56$ degrees of freedom are calculated for the differences in log-odds and the differences in mean pain score between the alternative treatment regimens B, C and D and the reference regimen A.

```
library(mmmgee)
data(keratosis)
m1<-geem2(clearance~trt,id=id,data=keratosis,family=binomial,
          corstr="independence")
m2<-geem2(pain~trt,id=id,data=keratosis[keratosis$lesion==1,],
          family=gaussian,corstr="independence")
L1<-L2<-diag(1,4)[-1,]
mmmgee.test(x=list(m1,m2),L=list(L1,L2),
        asymptotic=FALSE,biascorr=TRUE,conf.int=TRUE)
```

The resulting point estimates and simultaneous 95% confidence intervals are shown in Table S.2

Table S.2: *Point estimates and simultaneous 95% confidence intervals for the difference in log-odds of lesion clearance and the mean difference of pain scores between the standard regimen A and the alternative therapy regimens B, C and D in the actinic keratosis example.*

		Estimate	Lower	Upper
Lesion clearance				
	B vs A	0.08	-0.41	0.56
	C vs A	-0.27	-0.78	0.24
	D vs A	-1.07	-1.65	-0.49
Pain score				
	B vs A	-1.04	-1.32	-0.76
	C vs A	-1.14	-1.45	-0.82
	D vs A	-1.99	-2.27	-1.71

Confidence intervals for differences in proportions and mean differences

To calcualte intervals for the difference in proportions of lesion clearence rather than log-odds, the delta method is applied. This means to approximate the distribution of a differentiable function $\boldsymbol{f} = \boldsymbol{f}(\hat{\boldsymbol{\beta}})$ as $N(\boldsymbol{f}(\boldsymbol{\beta}), \frac{\partial \boldsymbol{f}(\hat{\boldsymbol{\beta}})}{\partial \boldsymbol{\beta}} \hat{var}(\hat{\boldsymbol{\beta}}) \frac{\partial \boldsymbol{f}(\hat{\boldsymbol{\beta}})}{\partial \boldsymbol{\beta}^T})$. In the example, the calculation is simplified if we use the no-intercept model

$$\log \frac{\mu_{ij}^{(1)}}{1-\mu_{ij}^{(1)}} = \mathbb{1}_{\{trt_{ij}=A\}}\beta_1^{(1)} + \mathbb{1}_{\{trt_{ij}=B\}}\beta_2^{(1)} + \mathbb{1}_{\{trt_{ij}=C\}}\beta_3^{(1)} + \mathbb{1}_{\{trt_{ij}=D\}}\beta_4^{(1)}$$

for the binary endpoint, such that $p_k = \exp(\beta_k^{(1)})/(1+\exp(\beta_k^{(1)}))$ is the probability of clearance in the k-th treatment group. Define $\boldsymbol{f}(\boldsymbol{\beta}) = (p_1, \dots, p_4, \beta_0^{(2)}, \dots, \beta_3^{(2)})^T$. Then $\frac{\partial \boldsymbol{f}(\hat{\boldsymbol{\beta}})}{\partial \boldsymbol{\beta}}$ is the diagonal matrix $diag(p_1(1-p_1), \dots, p_1(4-p_4), 1, 1, 1, 1)$. Simultaneous confidence intervals for contrasts of $\boldsymbol{f}(\boldsymbol{\beta})$ are then calculated based on the corresponding normal approximation of $\boldsymbol{f}(\hat{\boldsymbol{\beta}})$, analogous to the calculation of confidence intervals for contrasts of $\boldsymbol{\beta}$ as defined in equation (2) of the main manuscript.

The following R code is used to perform the calculations. Here the 'multcomp' package [4] is used to calculate the simultaneous confidence intervals based on the estimated proportions and means and the delta method estimate of their covariance matrix. The first line of code installs the 'multcomp' package. As before, the critical value from a multivariate

t-distribution with $K - 4 = 56$ degrees of freedom is used.

```
install.packages("multcomp")
library(mmmgee)
library(multcomp)
data(keratosis)
m1_noint<-geem2(clearance~trt-1,id=id,data=keratosis,
        family=binomial,init.beta=rep(0.5,4))
m2<-geem2(pain~trt,id=id,data=keratosis[keratosis$lesion==1,],
          family=gaussian,corstr="independence")
p<-exp(coef(m1_noint))/(1+exp(coef(m1_noint)))
G<-mmmgee(list(m1_noint,m2),biascorr=TRUE)
D<-diag(c( p*(1-p), rep(1,4) ))
Ldelta<-diag(1,8)[-c(1,5),]
Ldelta[1:3,1]<- -1
confint(glht(model=NULL,linfct=Ldelta,coef.=c(p,coef(m2)),
        vcov.=D%*%vcov(G)%*%D,df=60-4))
```

The resulting point estimates and simultaneous 95% confidence intervals are shown in Table S.3 In this example, the results would suggest that all alternative treatment regimens result in a reduction of pain. For treatment B the difference in lesion clearance proportion is in a reasonably close margin around zero, whereas for treament C and D a severe reduction on efficacy cannot be excluded.

Table S.3: *Point estimates and simultaneous 95% confidence intervals for the difference in proportion of lesion clearance and the mean difference of pain scores between the standard regimen A and the alternative therapy regimens B, C and D in the actinic keratosis example.*

	Estimate	Lower	Upper
Lesion clearance			
B vs A	0.02	-0.09	0.12
C vs A	-0.06	-0.18	0.05
D vs A	-0.26	-0.39	-0.12
Pain score			
B vs A	-1.04	-1.32	-0.76
C vs A	-1.14	-1.45	-0.82
D vs A	-1.99	-2.27	-1.71

References

[1] Lloyd A Mancl and Timothy A DeRouen. A covariance estimator for gee with improved small-sample properties. *Biometrics*, 57(1):126–134, 2001.

[2] Aad W Van der Vaart. *Asymptotic statistics*. Cambridge University Press, 2000.

[3] Robin Ristl. *mmmgee: Simultaneous Inference for Multiple Linear Contrasts in GEE Models*, 2019. URL `https://CRAN.R-project.org/package=mmmgee`. R package version 1.20.

[4] Torsten Hothorn, Frank Bretz, and Peter Westfall. Simultaneous inference in general parametric models. *Biometrical journal*, 50(3):346–363, 2008.
